# Neurotechnologies under the Eye of Bioethics

**DOI:** 10.1523/ENEURO.0072-22.2022

**Published:** 2022-06-16

**Authors:** Catherine Vidal

**Affiliations:** Honorary Research Director at the Pasteur Institute of Paris, Member of the Ethics Committee of the French Health Research Institute, Institut National de la Santé et de la Recherche Médicale

**Keywords:** brain data protection, mental privacy, neuroethics, neurorights, neurotechnologies

## Significance Statement

Neurosciences and digital technologies combine into the booming field of “neurotechnologies” (NT). Prospects for medical applications are very promising. If the GAFAM invest millions of dollars in NT, this is not only to develop brain-machine interfaces to overcome disabilities and mental pathologies. There are also commercial issues aimed at the public, via the exploitation of brain data for personal uses and for supervision of individual behaviors. The capacity of NT to “manipulate the brains” calls for vigilance in two particular areas: respect of mental autonomy and protection of brain data. A major issue is to assess whether existing laws on the protection of human rights are sufficient to protect mental privacy or whether new rights - NeuroRights- must be established specifically.

The present era is characterized by major advances in neurosciences and in digital technologies. Both combine into a booming field of investigation called “neurotechnologies” (NTs) that result from research into brain pathophysiology, miniaturization of electronic systems, and increasing capacities of computers to process digital data. Implanting microprocessors in the brain, decoding electrical signals produced by neurons, stimulating specific brain regions to modify their activity, are all now possible to be applied to the human brain. Such techniques are already being used successfully in compensating some physical and mental handicaps. The prospects for medical applications are very promising and constitute real progress in improving the quality of life for patients (Donoghue, 2015; Moritz et al., 2016; Vidal, 2019).

If the GAFAM (Google, Amazon, Facebook, Apple, and Microsoft) invest millions of dollars in NT, this is not only to develop brain-machine interfaces designed to overcome disabilities and mental pathologies. Beyond such laudable goals, there are commercial issues aimed at the public, via the exploitation of brain data for personal uses and for supervision of individual behaviors (Ienca et al., 2018; Koening, 2019; Kellmeyer, 2021).

A potentially very lucrative field of application of NT is what is now called “well-being therapies.” Startups are developing helmets and headbands equipped with electrodes to record electroencephalograms, with the aim of permitting self-control of stress, sleep, and concentration. It is noteworthy that brain wave recording technologies with electrodes placed on the skull are nothing new. It has been known for over 50 years that α frequency waves are associated with relaxation and β waves with alertness. What is new is the possibility of real-time processing data by smartphone applications. Each type of brain wave is translated into sound or visual signals that inform the user about their brain activity. They have to focus their attention on the signals and gradually learn to correlate them with their brain state: this is the “neuro-feedback” method. It should be stressed that until now no validation of the efficiency or the harmlessness of these devices has been demonstrated by any rigorous scientific method (Coates McCall et al., 2019; Wexler and Thibault, 2019). However, Apple and Samsung are already incorporating neurogadgets in their new smartphones to read brain activity. The GAFAM’s ambition is to develop new cloud infrastructures to store brain data and combine them with other personal data that they will take over to use for profit.

## Brain Supervision

Beyond health and personal well-being uses, other applications of NT are designed to monitor mental states in everyday life, to control them in case of failure. Thus, the driving of vehicles is already supervised by cameras and sensors that provide information about the driver's level of alertness. In a near future, it is highly probable that brain wave recording devices, supposed to be more reliable, will be used for this purpose. The automaker Nissan (https://www.nissan-global.com/EN/INNOVATION/TECHNOLOGY/ARCHIVE/B2V/) and the supplier Hyundai Mobis (https://www.youtube.com/watch?v=POKrrDrr1wM) are the most advanced in this field.

In China, brain supervision is a very concrete reality. In some factories, including one in Hangzhou city, the 40,000 workers wear headsets with brain wave sensors to monitor their concentration on assembly lines (https://www.thetimes.co.uk/article/china-chinese-read-brainwaves-workforce-technology-mood-detection-zp67vv9vx). The company’s profits are claimed to significantly increase thanks to the reassignment of distracted or stressed individuals to other tasks in the production chain.

In the field of education, devices aimed to control the behavior and brains of pupils are being tested at school. A growing number of classrooms are equipped with cameras linked to facial recognition software to monitor children’s behavior during lessons. Bluetooth bracelets record pupil’s heartrate and the time spent in the library and on playground (https://www.wsj.com/articles/chinas-efforts-to-lead-the-way-in-ai-start-in-its-classrooms-11571958181). Supervision of brain activities is also being tested. In some primary schools, pupils wore headsets to check their attention level in the classroom (https://www.theguardian.com/world/2019/nov/01/chinese-primary-school-halts-trial-of-device-that-monitors-pupils-brainwaves). Data were transmitted to the teacher’s computer, thus allowing the real-time assessment of pupil’s concentration and the detection of nonattentive students. A report was sent to the parents after the class. The claimed purpose was to improve school performance: support to the teacher and psychological pressure on the pupil to better concentrate. But it backlashed.

## The Ethical, Legal, and Societal Challenges of NTs

The NT market for nonmedical purposes is booming. This implies to examine closely the ethical and legal issues of the use of these technologies, which will affect an increasing audience. The capacity of NT to “manipulate the brains” calls for vigilance in two particular areas: respect of mental autonomy and protection of brain data (Ienca and Andorno, 2017; Yuste et al., 2017; Minielly et al., 2020; Rainey et al., 2020; Pfotenhauer et al., 2021):

1. Mental autonomy. Is the use of NT likely to make people more efficient and freer or can it be a source of enslavement and undermine mental integrity? There are many gray areas about the benefit/risk ratio of NT. For example, the hope of increasing one’s cognitive performance can lead to addictive behaviors, particularly in vulnerable subjects, in adolescents, and in the elderly. It should not be excluded that the prolonged use of NT might interfere with cerebral physiology at structural and functional levels. “Neuro-surveillance” in everyday life can lead to the stigmatization and discrimination of some people. The use of NT at the workplace raises questions in terms of labor rights for employees subjected to management guidelines (https://onlabor.org/neuro-surveillance-and-the-right-to-be-humans-at-work).

2. Protection of brain data. The collection, storage, and utilization of brain data by digital companies present the risk of being exploited by advertisers, insurance companies, police forces, etc. This exploitation might occur without the awareness of users who do not have sufficient information to give or not to give their consent. And even if consent is given, the risk is that information on a person’s brain characteristics might be used for another purpose than the one explicitly consented by the user. It is imperative to inform the public that brain waves are not “trivial” data such as heartrate or daily step count, which are now routinely recorded by smartphone applications. Data on brain functioning, combined with other personal data, can provide precise information about people’s behavior, personality, and their intimate mental lives (Ienca et al., 2018; Kellmeyer, 2021).

## Ensuring Privacy and Security of Brain Data

In response to the large development of NTs in many fields out of the medical and research contexts, the highest priority must be to guarantee the privacy and security of brain data. Existing regulatory policies have to be strengthened to lead NT companies and service providers to exercise much more transparency concerning the conditions of use, sharing, and anonymization of brain data. It is also necessary to improve consent procedures with complete and understandable information to allow citizens to have control over their data. These best practice measures should be put in place by both governmental regulatory bodies and private sector companies (Minielly et al., 2020; Pfotenhauer et al., 2021).

## Neurorights, an Emerging Category of Fundamental Rights

All technological devices that aim to modify, improve, and control the human brain challenge individual liberty. Debates on these topics have recently crystallized around the notion of “neurorights” such as the rights to mental privacy (to protect brain data against their unauthorized collection), to mental integrity (against harmful manipulation of brain activity), and cognitive liberty (freedom of thought and conscience; Ienca and Andorno, 2017; Yuste et al., 2017).

Topics of neuroethics and neurorights are the key points of several reports on NT recently published by the Organisation for Economic Co-operation and Development (OECD; https://www.oecd.org/sti/emerging-tech/recommendation-on-responsible-innovation-in-neurotechnology.htm), European Council (https://rm.coe.int/report-final-en/1680a429f3), and Unesco (https://unesdoc.unesco.org/ark:/48 223/pf0000378724).

A major issue is to assess whether existing laws on the protection of human rights are sufficient to protect mental privacy or whether new rights must be established specifically (Rainey et al., 2020; Ienca, 2021; Yuste et al., 2021). According to many lawyers and experts, these rights are not defended by existing legal protection. This situation has been already addressed by Chile, which in 2021 became the first country in the world to pass a law, included in the constitution, to protect brain data and prohibit their use without informed consent (https://en.unesco.org/courier/2022-1/chile-pioneering-protection-neurorights).

In France, the situation is still far from a recognition of neurorights (Vidal, 2020). In 2018, the National Consultative Ethics Committee (CCNE) took up the subject in the scope of the revision of the bioethics law initially scheduled for 2020. A bill recommended to supervise the technologies of brain activity recording, limiting their use to “medical and scientific research or forensic expertise,” with particular emphasis on the protection of brain data. The law finally passed in 2021, it includes these principles, but the question of the use and protection of brain data does not appear explicitly (https://www.legifrance.gouv.fr/jorf/id/jorftext000043884384).

Obviously, legislative developments are only progressing slowly compared with the rapid evolutions in the field of NTs. In this context, the North American foundation “NeuroRights” recommends that the UN plays a leading role to reach a worldwide consensus to protect neurorights (https://neurorightsfoundation.org/). Let’s hope that such a proposal will allow to effectively put in place the ethical framework of NT development for the benefit of technological innovation while respecting fundamental human rights.

## Synthesis

Reviewing Editor: Karl Herrup, University of Pittsburgh School of Medicine Decisions are customarily a result of the Reviewing Editor and the peer reviewers coming together and discussing their recommendations until a consensus is reached. When revisions are invited, a fact-based synthesis statement explaining their decision and outlining what is needed to prepare a revision will be listed below. The following reviewer(s) agreed to reveal their identity: NONE.

This is a timely and much needed work. Discussion of the ethical dimensions of the new technologies available in the realm of brain data is critical to have now, before the technology gets ahead of us as a society.

In that context, however, I would have hoped that the author would have emphasized a different message. The piece lists many examples of inroads into our private brain-data lives, but falls short in clearly identifying why these are ethical problems. It seems obvious at one level, but a clear outline of the ethical issues involved would be far more useful to the eNeuro readership than the detailed examples offered. If a consumer signs away their rights to their brain data (and who among us has ever really read the Terms of Use we all claim to have), why is there a problem. A clear delineation of two or three main ethical concepts that are at risk would offer a stronger focus to the commentary than the listing of the particular examples cited.

The author is encouraged to take this more philosophical approach to enhance the contribution of the submission.
